# DAPSA scores reflect both articular and cutaneous involvement in psoriatic arthritis^☆^

**DOI:** 10.1016/j.abd.2026.501368

**Published:** 2026-05-18

**Authors:** Qianzi Liu, Minjia Tan, Yehong Kuang

**Affiliations:** a Department of Dermatology, Xiangya Hospital, Central South University, Changsha, China; b National Engineering Research Center of Personalized Diagnostic and Therapeutic Technology, Changsha, China; c Furong Laboratory, Changsha, China; d National Clinical Research Center for Geriatric Disorders, Xiangya Hospital, Central South University, Changsha, China; e Hunan Key Laboratory of Skin Cancer and Psoriasis, Xiangya Hospital, Central South University, Changsha, China; f Hunan Engineering Research Center of Skin Health and Disease, Xiangya Hospital, Central South University, Changsha, China; g Xiangya Clinical Research Center for Cancer Immunotherapy, Central South University, Changsha, China

**Keywords:** Arthritis, psoriatic, Cross-sectional studies, Outcome assessment, health care, Skin diseases, Severity of illness index

## Abstract

**Background:**

The Disease Activity Index for Psoriatic Arthritis (DAPSA) is a widely used tool to assess joint involvement in Psoriatic Arthritis (PsA), but its ability to reflect skin disease severity remains unclear.

**Objective:**

This study aimed to investigate the association between DAPSA scores and skin disease severity in patients with PsA.

**Methods:**

This single-center, cross-sectional study included PsA patients meeting the CASPAR classification criteria at Xiangya Hospital from April 2019 to February 2025. Skin disease severity was assessed using the Psoriasis Area and Severity Index (PASI) and Dermatology Life Quality Index (DLQI), while disease activity was assessed with the DAPSA. Propensity Score Matching (PSM) was applied to adjust for musculoskeletal disease activity.

**Results:**

Among the 646 PsA patients (median age 46.0 years; 41.0% male), DAPSA scores increased from 12.6 (SD = 13.2) in patients with no skin disease (PASI = 0) to 20.4 (SD = 16.9) in those with severe skin disease (PASI > 10). Patient global assessment, patient pain assessment scores and C-reactive protein levels also rose with PASI severity (all p < 0.001). After PSM, patients with PASI ≥ 10 had significantly higher DAPSA scores than those with PASI < 10 (25.4 vs. 16.9, p < 0.001). A weak but statistically significant positive correlation was observed between DAPSA and PASI scores (Spearman's rho = 0.256, p = 0.003).

**Study limitations:**

Cross-sectional design, single-center setting and potential residual confounding limit causal inference and generalizability.

**Conclusion:**

Although not designed to assess skin disease, DAPSA may partially capture skin-related burden through its inflammatory and patient-reported components. In the absence of dedicated skin assessments, DAPSA could serve as a practical and holistic tool for initial disease activity evaluation in PsA.

## Introduction

Psoriatic Arthritis (PsA) is a chronic inflammatory disease that affects approximately 20%‒30% of patients with psoriasis.^1^ It is characterized by heterogeneous manifestations, including peripheral arthritis, axial involvement, enthesitis, dactylitis, and cutaneous disease.^2^ PsA can lead to irreversible joint damage, functional disability, and reduced quality of life.^3^ The complexity of PsA poses significant challenges in comprehensively assessing disease activity, necessitating the use of composite measures that integrate multiple clinical domains.

Among the composite indices developed to assess disease activity in PsA, the Disease Activity Index for Psoriatic Arthritis (DAPSA) has emerged and shown high sensitivity and specificity in distinguishing disease states.^4^ It incorporates Tender Joint Counts (TJC), Swollen Joint Counts (SJC), Patient Global Assessment (PtGA), Patient Pain Assessment (PtPA), and C-Reactive Protein (CRP), providing a quantitative and clinically feasible assessment of musculoskeletal disease activity.^4^, ^5^ Previous studies have demonstrated the strong correlation between DAPSA and ultrasound assessments and their predictive value for radiographic progression.^6^ Furthermore, compared with the complex calculation method of Psoriatic Arthritis Disease Activity Score (PASDAS),^7^, ^8^ the DAPSA requires only five variables for computation, making it widely applicable in both clinical practice and research settings.^9^, ^10^, ^11^

Although DAPSA effectively captures articular involvement, its ability to reflect extra-articular manifestations, particularly the severity of skin disease, remains uncertain. Skin disease severity, typically assessed using the Psoriasis Area and Severity Index (PASI) or Dermatology Life Quality Index (DLQI), may influence PtGA Visual Analogue Scale (VAS) scores^12^ and CRP,^13^, ^14^, ^15^, ^16^, ^17^ which are key components of DAPSA. Nevertheless, the extent to which DAPSA indirectly reflects skin disease activity through its various components warrants further investigation.

Therefore, the present study aimed to evaluate whether DAPSA reflects skin disease severity in PsA patients in a Chinese cohort. Specifically, the authors examined the associations between DAPSA and established measures of skin severity (PASI and DLQI) and assessed whether skin involvement affects patient-reported components of the DAPSA score. Understanding this relationship is crucial for interpreting DAPSA scores in patients with prominent skin disease and may inform future approaches to comprehensive PsA assessment.

## Method

### Study design and participants

This single-center, cross-sectional study was conducted at the Dermatology Department of Xiangya Hospital, Central South University in China, and included patients with PsA who met the CASPAR classification criteria^18^ between April 2019 and February 2025. The diagnosis of PsA was confirmed by an experienced dermatologist and/or rheumatologist. Written informed consent was obtained from all participants. The study adhered to the Declaration of Helsinki and Good Clinical Practice guidelines. Ethical approval for this study was granted by the Ethics Committees of Xiangya Hospital of Central South of University (approval number: 2018121106).

### Data collection

Baseline demographic and clinical characteristics were obtained from electronic medical records, including age, sex, duration of psoriasis, and duration of PsA. Additionally, the following clinical parameters were collected: PtGA, PtPA, 68 TJC, 66 SJC, enthesitis and dactylitis counts, and CRP levels (mg/L). PtGA and PtPA were measured using a Visual Analog Scale (VAS) ranging from 0 to 10 cm. DAPSA was calculated as: TJC + SJC + PtGA + PtPA + CRP (mg/dL).^4^ PASI scores were categorized as none (0), mild (< 3), moderate (3–10), or severe (> 10), while DLQI scores were categorized as having no impact (0–1), small impact (2–5), moderate impact (6–10), large impact (11–20), or extreme impact (21–30). Other covariates collected included the Physical Component Summary score of the 36-Item Short Form Survey (SF-36 PCS) and the Health Assessment Questionnaire (HAQ) score. Due to incomplete clinical assessments (such as joint counts, laboratory tests, or skin evaluations) in some patients, sample sizes varied across different variables. All available data were included in the analysis without imputation of missing values to preserve the authenticity of the findings.

### Statistical analysis

For statistical analysis, continuous variables were summarized as median (Interquartile Range, IQR) due to non-normal distributions, while categorical variables were presented as number (%). The association between DAPSA and PASI categories was compared using ANOVA, and Spearman correlation coefficients were calculated to assess the relationships between DAPSA and PASI. To control for confounding by musculoskeletal activity, Propensity Score Matching (PSM) was performed, grouping patients based on PASI ≥ 10 (severe skin involvement) versus PASI < 10 (non-severe skin involvement), with 1:1 matching conducted based on TJC, SJC, enthesitis, and dactylitis. The Mann-Whitney *U* test was used to compare outcomes between the matched groups. A sensitivity analysis was also performed by repeating the PSM for patients with moderate skin involvement (PASI 3–10) versus those with mild or no skin involvement (PASI < 3) to validate the consistency of the results. All analyses were conducted using SPSS v27.0, with a two-tailed p-value of less than 0.05 considered statistically significant.

## Results

### Patient characteristics

The study included 646 patients with PsA. The mean age of the cohort was 46.0 years (IQR: 36.0–55.0), with 41.0% being female. The median duration of psoriasis was 8.0 years (IQR: 3.0–15.0), and the median duration of PsA was 2.0 years (IQR: 0.5–4.5). The median DAPSA score was 14.4 (IQR: 8.3–24.0), indicating moderate disease activity. The median PASI score was 4.3 (IQR: 1.8–8.9), with 9.0% of patients having no psoriasis (PASI = 0), 26.3% having mild disease (PASI < 3), 42.5% having moderate disease (PASI 3–10), and 22.2% having severe disease (PASI > 10). The median DLQI score was 5.0 (IQR: 2.0–9.0), reflecting a moderate impact on quality of life (Table 1).

**Table 1:** Baseline demographics and clinical characteristics.

	Range	Median (IQR) or number (%)	n
**Age**		46.0 (36.0‒55.0)	646
**Female, n (%)**		265 (41.0)	646
**Duration of psoriasis (y)**		8.0 (3.0‒15.0)	646
**Duration of PsA (y)**		2.0 (0.5‒4.5)	643
**PtGA VAS**	0‒100	50.0 (20.0‒70.0)	641
**PtPA VAS**	0‒100	30.0 (20.0‒60.0)	641
**SJC**	0‒66	1.0 (0.0‒4.0)	636
**TJC**	0‒68	2.0 (1.0‒5.0)	636
**Enthesitis**	0‒6	0 (0‒0)	646
**Dactylitis**	0‒20	0 (0‒1.0)	616
**SF-36 PCS**	0‒100	70.6 (43.8‒82.3)	606
**HAQ**	0‒ 3	0 (0‒0.1)	604
**CRP (mg/L)**	0‒500	4.3 (1.6‒13.1)	600
**PASI**	0‒72	4.3 (1.8‒8.9)	607
**PASI, n (%)**			607
	No psoriasis	55 (9.0)	
	< 3	160 (26.3)	
	3‒10	259 (42.5)	
	> 10	135 (22.2)	
**DLQI**	0‒30	5.0 (2.0‒9.0)	556
**DAPSA**		14.4 (8.3‒24.0)	590

* Data are median (IQR) or n (%) unless otherwise stated.

IQR, Interquartile Range; PtGA, Patient Global Assessment; PtPA, Patient Pain Assessment; VAS, Visual Analogue Scale (0‒100); SJC, Swollen Joint Count; TJC, Tender Joint Count; SF-36 PCS, SF-36 Physical Component Summary score; HAQ, Health Assessment Questionnaire score; CRP, C-Reactive Protein; PASI, Psoriasis Area and Severity Index; DLQI, Dermatology Life Quality Index; DAPSA, Disease Activity index for PSoriatic Arthritis.

### DAPSA across skin disease activity scores

Table 2 presents the mean DAPSA, PtGA, PtPA scores, and CRP levels across subgroups stratified by skin disease severity, as measured by PASI and DLQI. DAPSA scores showed a significant upward trend with increasing severity of skin involvement. For PASI categories, DAPSA scores ranged from 12.6 (SD = 13.2) in patients with no skin disease (PASI = 0) to 20.4 (SD = 16.9) in those with severe skin disease (PASI > 10). Similarly, for DLQI categories, DAPSA scores ranged from 10.6 (SD = 10.2) in patients with no impact on quality of life (DLQI 0–1) to 29.5 (SD = 14.4) in those with an extreme impact (DLQI 21–30). PtGA, PtPA scores, and CRP levels exhibited an upward trend in parallel with increasing skin disease severity (all p-values < 0.001).

**Table 2:** DAPSA, PtGA scores, PtPA scores and CRP levels by PASI and DLQI categories.

	DAPSA	PtGA VAS	PtPA VAS	CRP
**PASI**	**(n = 590)**	**(n = 641)**	**(n = 641)**	**(n = 572)**
0 (none)	12.6 (13.2)	35.7 (30.3)	28.3 (30.3)	8.2 (22.0)
< 3 (mild)	15.9 (12.8)	43.2 (27.6)	36.6 (28.4)	7.7 (14.6)
3-10 (moderate)	18.9 (13.7)	52.8 (25.4)	41.9 (27.0)	13.2 (24.1)
> 10 (severe)	20.4 (16.9)	63.8 (23.9)	40.6 (27.4)	23.3 (34.7)
**DLQI**				
0‒1 (none)	10.6 (10.2)	33.9 (28.8)	25.6 (27.5)	9.8 (17.7)
2‒5 (small)	17.3 (13.9)	48.9 (24.4)	36.5 (26.4)	13.8 (28.4)
6‒10 (moderate)	20.1 (16.9)	54.5 (26.2)	44.6 (27.5)	12.9 (24.3)
11‒20 (large)	23.2 (15.1)	66.5 (23.2)	47.0 (27.6)	19.3 (30.8)
21‒30 (extreme)	29.5 (14.4)	72.9 (31.5)	65.7 (27.6)	34.3 (52.9)

DAPSA, Disease Activity index for PSoriatic Arthritis; PASI, Psoriasis Area and Severity Index; DLQI, Dermatology Life Quality Index; PtGA, Patient Global Assessment; PtPA, Patient Pain Assessment; VAS, Visual Analogue Scale (0‒100); CRP, C-Reactive Protein.

### Propensity score matching analysis

To further evaluate the impact of skin disease severity on DAPSA, patients were matched for musculoskeletal disease activity using propensity scores. In the matched cohort (n = 178, patients with severe skin involvement (PASI ≥ 10) had significantly higher DAPSA scores (25.4 vs. 16.9, p < 0.001), PtGA (73.4 vs. 49.6, p < 0.001), PtPA (50.4 vs. 37.9, p = 0.002), CRP (28.1 vs. 8.5, p < 0.001) and DLQI scores (9.0 vs. 5.2, p < 0.001) compared to those with mild to moderate skin involvement (PASI < 10) (Table 3).

**Table 3:** Comparison of groups matched for severity of musculoskeletal disease.

	PASI ≥ 10	PASI < 10	p-value
	(n = 89)	(n = 89)	
**Age**	46.2 (12.3)	45.5 (14.3)	0.753
**Psoriasis duration(y)**	10.9 (9.7)	10.4 (11.4)	0.732
**PsA duration(y)**	4.0 (4.8)	3.8 (5.8)	0.890
**PtGA VAS**	73.4 (15.9)	49.6 (28.5)	<0.001
**PtPA VAS**	50.4 (23.7)	37.9 (30.1)	0.002
**SJC**	4.7 (8.0)	3.0 (4.7)	0.080
**TJC**	5.5 (7.9)	4.3 (7.8)	0.314
**Enthesitis**	0.2 (0.7)	0.2 (0.9)	0.924
**Dactylitis**	1.5 (3.6)	1.2 (2.6)	0.508
**SF-36 PCS**	51.6 (22.0)	62.7 (22.5)	0.002
**HAQ**	0.3 (0.5)	0.2 (0.5)	0.353
**CRP (mg/L)**	28.1 (39.0)	8.5 (14.0)	<0.001
**DLQI**	9.0 (5.0)	5.2 (4.1)	<0.001
**DAPSA**	25.4 (17.4)	16.9 (13.2)	<0.001

PsA, Psoriatic Arthritis; PtGA, Patient Global Assessment; PtPA, Patient Pain Assessment; VAS, Visual Analogue Scale (0‒100); SJC, Swollen Joint Count; TJC, Tender Joint Count; SF-36 PCS, SF-36 Physical Component Summary score; HAQ, Health Assessment Questionnaire score; CRP, C-Reactive Protein; PASI, Psoriasis Area and Severity Index; DLQI, Dermatology Life Quality Index; DAPSA, Disease Activity index for PSoriatic Arthritis.

A sensitivity analysis comparing patients with moderate skin involvement (PASI 3–10) to those with mild or no involvement (PASI < 3) revealed similar trends, with higher PtGA (55.3 vs. 40.7, p < 0.001), PtPA (42.5 vs. 34.2, p = 0.005), CRP (15.6 vs. 8.21, p < 0.001) and DLQI scores (6.6 vs. 4.7, p = 0.001). DAPSA scores remained significantly elevated in the moderate skin involvement group (19.2 vs. 15.7, p = 0.025) (Supplementary Table S1).

### Correlation between DAPSA and PASI

A scatterplot of DAPSA and PASI scores demonstrated a weak but statistically significant positive correlation (Spearman’s rho = 0.256, p = 0.003) (Supplementary Fig. S1). ANOVA analysis further confirmed the association, showing a significant difference in DAPSA scores across PASI categories (*F* = 4.917, p = 0.002) (Supplementary Fig. S2).

## Discussion

Musculoskeletal and cutaneous manifestations in PsA are often thought to progress independently,^19^ making comprehensive disease assessment challenging. Existing tools largely focus on isolated disease domains and may overlook the full clinical spectrum of PsA.^10^ In the cross-sectional study of 646 Chinese patients with PsA, the authors found that DAPSA, although originally developed for peripheral joint assessment, was significantly associated with skin disease severity. This may be attributed to its inclusion of PtGA, PtPA scores, and CRP levels, which capture elements of systemic inflammation and patient-perceived disease burden. These findings suggest that DAPSA may reflect not only musculoskeletal involvement but also cutaneous manifestations, offering a more holistic measure of PsA disease activity.

The observed increase in PtGA and PtPA scores with worsening PASI supports the hypothesis that skin disease severity contributes to patient-reported outcomes. These findings are aligned with prior studies reporting significant correlations between patient-reported global assessments and PASI,^20^, ^21^, ^22^ helping to explain why DAPSA, despite lacking a formal skin domain, can indirectly reflect cutaneous involvement. Moreover, even after matching for musculoskeletal disease activity using PSM, patients with more severe skin involvement exhibited higher DAPSA scores. Notably, patients with severe skin involvement (PASI ≥ 10) had significantly elevated DAPSA scores, global assessments, and CRP levels compared to those with milder skin disease (PASI < 10). These findings underscore the potential role of systemic inflammation, as reflected by elevated CRP, in linking articular and cutaneous manifestations. This association is further supported by previous studies demonstrating that PsA patients with higher CRP levels tend to have more severe skin disease.^13^, ^14^, ^15^, ^16^, ^17^

Previous studies have demonstrated the validity of DAPSA, showing strong correlations with ACR response and Minimal Disease Activity (MDA), with high sensitivity and specificity (both ≥ 90%), including when compared with joint ultrasonography.^5^, ^23^, ^24^, ^25^, ^26^, ^27^ The present study is the first to examine the relationship between DAPSA and skin disease severity. The authors observed a weak correlation between DAPSA and PASI, which should be interpreted cautiously. DAPSA should not be considered a substitute for skin assessments, particularly in patients with severe cutaneous involvement. Its practical utility as a holistic screening tool may be best suited for initial global assessment in settings where formal dermatology scoring is unavailable, rather than for precise quantification of skin activity.

Several limitations should be acknowledged. First, the cross-sectional design limits our ability to establish causal relationships between DAPSA and skin disease severity. Second, this study was conducted at a single tertiary center in China, which may limit the generalizability of the present findings to other populations with different genetic, environmental, and clinical characteristics. Thirdly, although the authors applied propensity score matching to adjust for differences in musculoskeletal disease activity, residual confounding due to unmeasured variables ‒ such as psychological burden, treatment adherence, or subclinical inflammation ‒ may still exist. These limitations highlight the need for well-designed prospective studies to clarify the dynamic interplay between skin and joint involvement in PsA and to refine integrated disease activity measures.

Clinically, the present results highlight that DAPSA, although not a substitute for dedicated skin assessments, may serve as a practical and holistic tool for initial disease activity evaluation in PsA, particularly where PASI or DLQI are unavailable. Future research should explore longitudinal relationships between DAPSA and skin severity, and whether integrating PASI into composite indices improves their predictive validity for outcomes like treatment response or radiographic progression.

## ORCID IDs

Qianzi Liu: 0009-0000-8166-6186

Minjia Tan: 0000-0002-8250-8216

## IRB approval status

Reviewed and approved by Xiangya Hospital (approval nº 2018121106).

## Financial support

This research was funded by the National Key Research and Development Program of China (2023YFC2508105), 10.13039/501100001809National Natural Science Foundation of China (82573986, 82373484, 82003354, 82221002, 82130090,82003362).

## Authors' contributions

Qianzi Liu: Methodology; formal analysis; writing-original draft.

Minjia Tan: Conceptualization; data curation; writing-review and editing.

Yehong Kuang: Supervision; funding acquisition; writing, review, and editing.

Qianzi Liu and Minjia Tan share the leading authorship of this manuscript because of their equal and significant contributions to this study.

## Research data availability

The entire dataset supporting the results of this study was published in this article.

## Conflicts of interest

None declared.
