# Supplementary material for: DAPSA scores reflect both articular and cutaneous involvement in psoriatic arthritis
Source: An Bras Dermatol. 2026 May 18;101(3):501368. doi: 10.1016/j.abd.2026.501368 (PMC13202544; doi:10.1016/j.abd.2026.501368)

ABD-D-25-00959_Supplementary Material

**Supplementary Table S1** Comparison of matched groups using the 3‒10 PASI cohort.

|  | **PASI  3‒10 (n = 183)** | **PASI < 3 (n = 183)** | **p-value** |
| --- | --- | --- | --- |
| **PtGA VAS** | 55.3 (23.1) | 40.7 (29.0) | <0.001 |
| **PP VAS** | 42.5 (26.7) | 34.2 (29.4) | 0.005 |
| **SJC** | 3.1 (4.4) | 3.3 (6.5) | 0.678 |
| **TJC** | 4.9 (7.7) | 4.2 (8.1) | 0.367 |
| **Enthesitis** | 0.2 (0.8) | 0.1 (0.5) | 0.193 |
| **Dactylitis** | 1.2 (2.9) | 1.3 (3.4) | 0.633 |
| **CRP (mg/L)** | 15.6 (27.4) | 8.2 (18.0) | <0.001 |
| **DLQI** | 6.6 (4.8) | 4.7 (4.9) | 0.001 |
| **DAPSA** | 19.2 (13.3) | 15.7 (16.4) | 0.025 |
| **HAQ** | 0.2 (0.4) | 0.5 (4.4) | 0.336 |

PhGA, Physician Global Assessment; PtGA, Patient Global Assessment; VAS, Visual Analogue Scale (0‒100); SJC, Swollen Joint Count; TJC, Tender Joint Count; SF-36 PCS, SF-36 Physical Component Summary score; HAQ, Health Assessment Questionnaire score; DLQI, Dermatology Life Quality Index; DAPSA, Disease Activity index for PSoriatic Arthritis.

**Supplementary Figure S1** Scatterplot of DAPSA and PASI. The spearman rho correlation coefficient was 0.256 (p = 0.003).


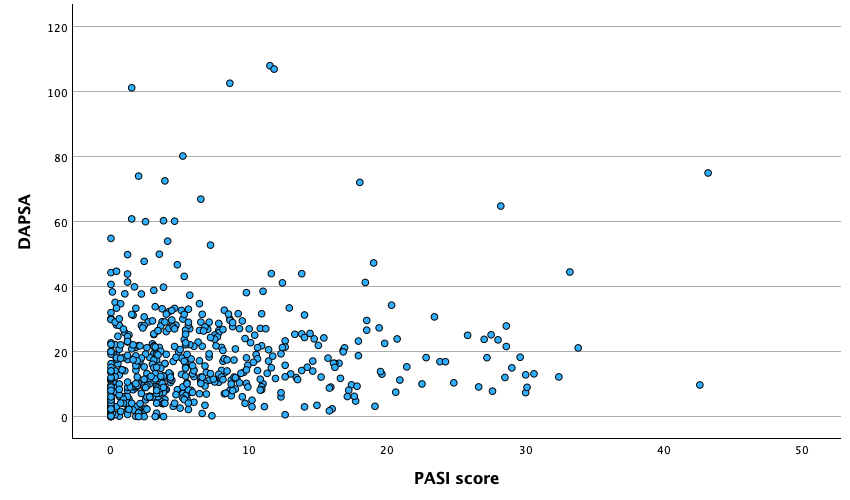


**Supplementary Figure S2** Boxplot of DAPSA by category of PASI. (0) No psoriasis, (1) PASI < 3, (2) PASI 3‒10; (3) PASI ≥ 10. ANOVA: *F* = 4.917, p = 0.002.


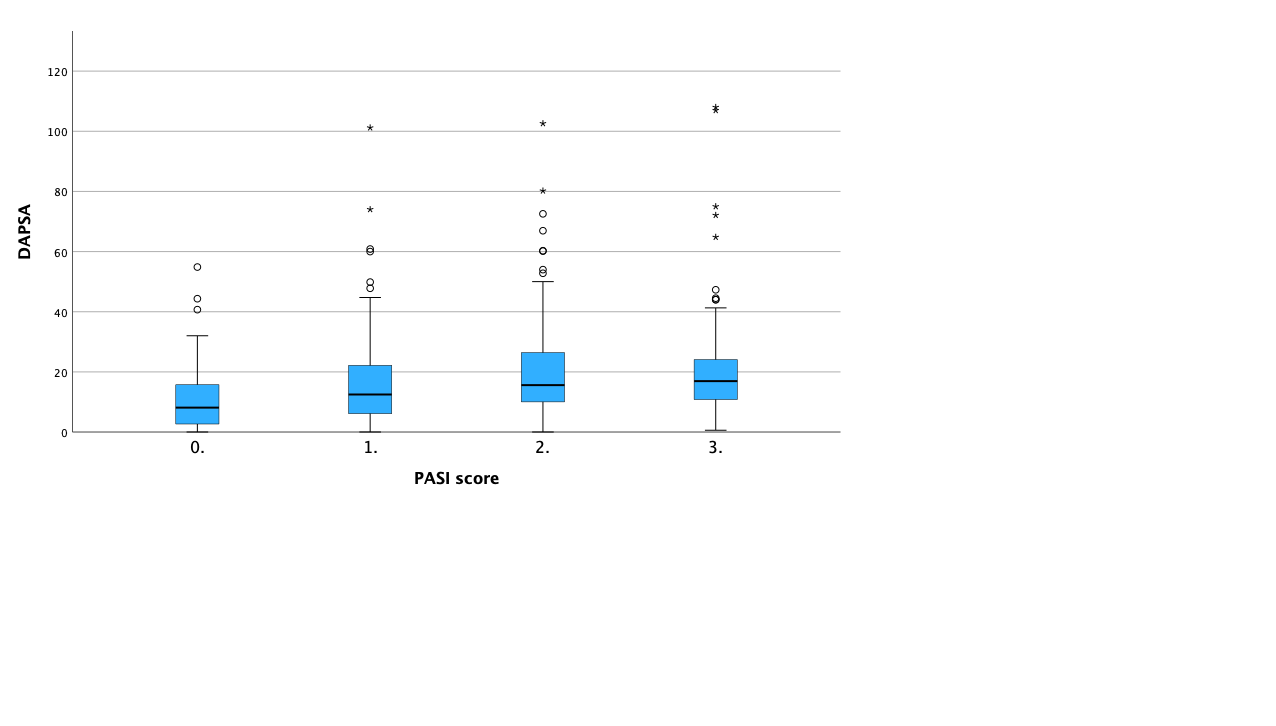

Supplement: Supplementary file 1 [file mmc1.docx]
